# Influencing factors of hospitalization cost of hypertension patients in traditional Chinese medicine hospitals

**DOI:** 10.3389/fpubh.2024.1329768

**Published:** 2024-04-25

**Authors:** Hao-jia Hou, Tian-zhen Cong, Yu Cai, Ya-hui Ba, Meng-en Chen, Jing-yu Yang, Zhong-hua Luo

**Affiliations:** ^1^School of Public Health, Gansu University of Chinese Medicine, Lanzhou, China; ^2^School of Business and Management, Gansu University of Chinese Medicine, Lanzhou, China; ^3^School of Traditional Chinese Medicine, Beijing University of Chinese Medicine, Beijing, China; ^4^School of Marxism Studies, Gansu University of Chinese Medicine, Lanzhou, China

**Keywords:** hypertension, TCM hospitals, characteristic advantages of TCM, hospitalization cost, length of stay

## Abstract

**Objectives:**

This study aimed to analyze the influencing factors of hospitalization cost of hypertensive patients in TCM (traditional Chinese medicine, TCM) hospitals, which can provide a scientific basis for hospitals to control the hospitalization cost of hypertension.

**Methods:**

In this study, 3,595 hospitalized patients with a primary diagnosis of tertiary hypertension in Tianshui City Hospital of TCM, Gansu Province, China, from January 2017 to June 2022, were used as research subjects. Using univariate analysis to identify the relevant variables of hospitalization cost, followed by incorporating the statistically significant variables of univariate analysis as independent variables in multiple linear regression analysis, and establishing the path model based on the results of the multiple linear regression finally, to explore the factors influencing hospitalization cost comprehensively.

**Results:**

The results showed that hospitalization cost of hypertension patients were mainly influenced by length of stay, age, admission pathways, payment methods of medical insurance, and visit times, with length of stay being the most critical factor.

**Conclusion:**

The Chinese government should actively exert the characteristics and advantages of TCM in the treatment of chronic diseases such as hypertension, consistently optimize the treatment plans of TCM, effectively reduce the length of stay and steadily improve the health literacy level of patients, to alleviate the illnesses pain and reduce the economic burden of patients.

## 1 Introduction

Hypertension is a common chronic cardiovascular disease, with persistently high blood pressure, causes damage to the heart, cerebral blood vessels, kidneys, and other organs, and can increase the risk of heart disease, stroke, and other diseases (1). By category, hypertension can typically be divided into essential hypertension, secondary hypertension, and pregnancy-induced hypertension (2). Hypertension has been identified as an essential risk factor for death, with an estimated 9.4 million premature deaths and 92 million disabilities attributable to hypertension each year (3–5). Based on available surveys, more than 1 billion people worldwide with hypertension in 2019, the number has doubled since 1990 (6). The largest global study of hypertension trends to date, led by Imperial College London and the World Health Organization in 2021 and involving more than 1,100 doctors and scientists, found the number of adults aged 30–79 with hypertension has increased from 650 million to 1.28 billion in the last 30 years and more than 700 million of these don't know they have (7). By 2019, China managed about 109 million registered hypertensive patients, the standardized management rate of hypertensive patients has increased by 29.28%, and the blood pressure control rate in the population managed for hypertension has increased by 16.84% in the past decade, with an average annual growth rate of 3.28% (8). In addition to the high prevalence of hypertension, the financial burden of this disease is also significant. The global healthcare costs due to hypertension exceed $500 billion per year, and only the United States incurs more than $300 billion per year, with Europe being the region with the highest healthcare costs for hypertension (9, 10). China's direct medical costs due to hypertension from 1993 to 2003 grew from RMB 4.531 billion to RMB 30.030 billion, with an average annual growth rate as high as 20.82%, which is faster than the GDP, and even faster than the growth rate of the total health costs and the total medical costs in the same period (11). The annual per capita cost of hypertension among Chinese residents in the period of 2006–2011 was RMB 6,271.80, accounting for 45.58% of the annual per capita income (12), and the medical cost of hypertension in China in 2019 was as high as RMB 74.06 billion, accounting for 5.1% of the total national medical cost (13, 14). With the aging of China's population, China's hypertensive population is bound to increase continually, and the hypertensive disease burden will continue to increase. Hypertension has become a worldwide public health problem bringing a heavy economic burden of disease to the world (15–17), and hypertensive patients with other comorbidities and complications will consume more medical resources and incur more healthcare costs (18, 19), so the need for research on hypertension cost control is particularly urgent (20–22).

As an essential part of China's healthcare system, TCM hospitals make comprehensive use of the correlation of all four examinations as well as traditional resources and therapeutic means, such as traditional Chinese medicine, acupuncture and moxibustion, and tuina, to carry out diagnosis and treatment for diseases. The TCM hospitals' therapy process emphasizes mutually the overall concept and individualized diagnosis, which focuses on regulating the balance and coordination of the systems within the human body while formulating targeted treatment plans according to the patient's specific condition and physical characteristics. Meanwhile, TCM hospitals inherit and carry forward the essence of TCM, as well as actively introduce modern medical technology, and promote the development of integrated Chinese medicine and Western medicine diagnosis and treatment modes, to satisfy the demand for patients' all-around diagnosis and treatment service. In recent years, the Chinese government has paid more and more attention to the inheritance and innovation of Chinese medicine and focused on the dominant therapy of TCM in the treatment of chronic diseases, such as cupping, scraping, acupuncture, and tuina (23–25). In particular, the publication of “Expert Consensus on the Diagnosis and Treatment of Hypertension with Traditional Chinese Medicine” (26, 27) has further improved the system of diagnostic and therapeutic protocols for the treatment of hypertension with Chinese medicine in China. Undeniably, TCM tonics, oral CPMs (Chinese patent medicines, CPMs), and TCM injections have been widely used in the treatment of hypertension and have achieved remarkable efficacy (28–30), which reflects unique TCM advantages in improving clinical efficacy, reducing BP levels, and improving the quality of life. In a word, TCM provides an excellent alternative for hypertensive patients who cannot tolerate conventional Western medications.

The Global Report on Hypertension released by WHO in September 2023, estimates the economic benefits of improved hypertension treatment options are ~18 times greater than the costs. As an important treatment method for hypertension in China, Chinese medicine, including herbal formulas, acupuncture, acupoints, footbaths, and other Chinese medical treatments, as well as the combination of Chinese medicine and Western medicine, not only achieve effective clinical results in the treatment of hypertension but also have an absolute advantage in terms of cost. In this regard, the overall cost of treating hypertension in TCM hospitals is much lower than Western medicine due to the vast area of cultivation of Chinese herbal medicine in China and the high availability and circulation of Chinese medicine resources. Therefore, this paper reviewed and analyzed the relevant information of 3,595 patients with hypertension who were hospitalized in Tianshui Hospital of TCM in Gansu Province, China from January 2017 to June 2022, to explore the influencing factors of hospitalization cost of patients. In this study, targeted countermeasures and suggestions were also proposed to control hospitalization cost and give full play to the price advantages of chronic diseases in TCM, to effectively alleviate the economic pressure brought by hypertension to the national economy.

## 2 Materials and methods

### 2.1 Study design and population

The data for this study came from the National Health Big Data Platform (a governmental non-full public database) of the Health Commission of Gansu Province, China, and the data content mainly involved information on the front page of TCM hospital cases, with detailed on hypertensive hospitalized patients in Tianshui City TCM Hospital, Gansu Province, for January 2017–June 2022. Inclusion criteria: patients with a primary diagnosis of tertiary hypertension I10.x05 (in accordance with ICD-10). Exclusion criteria: the length of stay is < 1 day, logically inconsistent in medical information, and diagnostic information with incomplete and could not be effectively supplemented. Following the above inclusion and exclusion criteria, 3,595 valid cases were finally included. As the research data need to be treated confidentially and should not be made public, the corresponding author can be contacted if necessary.

### 2.2 Data processing

Since the key indicator of hospitalization cost in this study is economic data, to eliminate the bias effect of inflation and other factors on the study of hospitalization cost in hypertension, the cost adjustment was made based on the Consumer Price Index (CPI) for healthcare in Gansu Province from 2017 to 2022, with 2016 as the base period for statistical correlation analysis.

According to the existing research, hospitalization cost is affected by factors such as length of stay (31–34). In this study, the length of stay and hospitalization cost were endogenous variables, while gender, ethnicity, age, marital status, visit times, payment methods of medical insurance, occupations, admission pathways, treatment categories, clinical pathways, use of TCM preparations, use of TCM diagnosis and treatment equipment, use of TCM diagnosis and treatment techniques, disease severity, and surgery and procedures were exogenous variables. In addition, relevant variables should be classified or integrated according to the actual analysis. The specific variable assignment processing was shown in Table 1.

**Table 1:** Variable assignment processing case.

**Variables**	**Variable codes**	**Variable names**	**Dummy variables**	**Variable assignment**
Endogenous variables	Y_1_	Length of stay (days)	—	Log (length of stay)
	Y_2_	Hospitalization cost (CNY¥)	—	Log (hospitalization cost)
Exogenous variables	X_1_	Gender	—	0 = male, 1 = female
	X_2_	Nationality	—	0 = han, 1 = other ethnic groups
	X_3 − 0_-X_3 − 2_	Age (years)	< 45 (reference)	0, 0
			45–60	1, 0
			>60	0, 1
	X_4_	Marital status	—	0 = married, 1 = others (unmarried, widowed or divorced)
	X_5_	Visit times	—	0 = one time, 1 = two or more times
	X_6 − 0_-X_6 − 3_	Payment methods of medical insurance	UEBMI^a^ (reference)	0, 0, 0
			URBMI^b^	1, 0, 0
			NCMS^c^	0, 1, 0
			Others	0, 0, 1
	X_7_	Occupations	—	0 = retired personnel, 1 = non-retired personnel
	X_8 − 0_-X_8 − 2_	Admission pathways	Emergency (reference)	0, 0
			Outpatients	1, 0
			Others	0, 1
	X_9 − 0_-X_9 − 2_	Treatment categories	—	0 = Chinese medicine 1 = integrated Chinese and western medicines
	X_10 − 0_-X_10 − 2_	Clinical Pathways	Chinese medicine (reference)	0, 0
			Western medicine	1, 0
			No clinical pathway	0, 1
	X_11_	Use of TCM^d^ preparations	—	0 = no, 1 = yes
	X_12_	Use of TCM^d^ diagnosis and treatment equipment	—	0 = no, 1 = yes
	X_13_	Use of TCM^d^ diagnosis and treatment techniques	—	0 = no, 1 = yes
	X_14_	Disease severity	—	0 = non-extremely high risk, 1 = extremely high risk
	X_15_	Surgery and procedures	—	0 = no, 1 = yes

^a^UEBMI, the urban employee basic medical insurance system; ^b^URBMI, the urban residents' basic medical insurance system; ^c^NCMS, the new cooperative medical scheme; ^d^TCM, traditional Chinese medicine.

### 2.3 Statistical analysis

Firstly, univariate analysis of length of stay and hospitalization cost was performed using SPSS 26.0. Since the original data of hospitalization cost and length of stay did not follow the normal distribution, the *Mann-Whitney U*-test and *Kruskal-Wallis H*-test were used to process and analyze according to the data types, and the length of stay and hospitalization cost were expressed in the median and quartile. Secondly, variables that were statistically significant in the univariate analysis selected as independent variables, and the dependent variables length of stay and hospitalization cost, which did not obey normal distribution, were logarithmically transformed into Log (Length of stay) and Log (Hospitalization cost) to approximate the requirement of obeying normality, to establish multiple linear regression models. Based on multiple linear regression analysis model, Amos 24.0 software was used to establish a path model with statistically significant variables in multiple linear regression results as independent variables, Log (Length of stay) as mediator variables, and Log (Hospitalization cost) as dependent variables, to explore the influencing factors and its rank relationship of hospitalization cost in patients with hypertension comprehensively. The test level of the above analysis was α = 0.05.

## 3 Results

### 3.1 Univariate analysis results of length of stay and hospitalization cost

**Figure 1:**
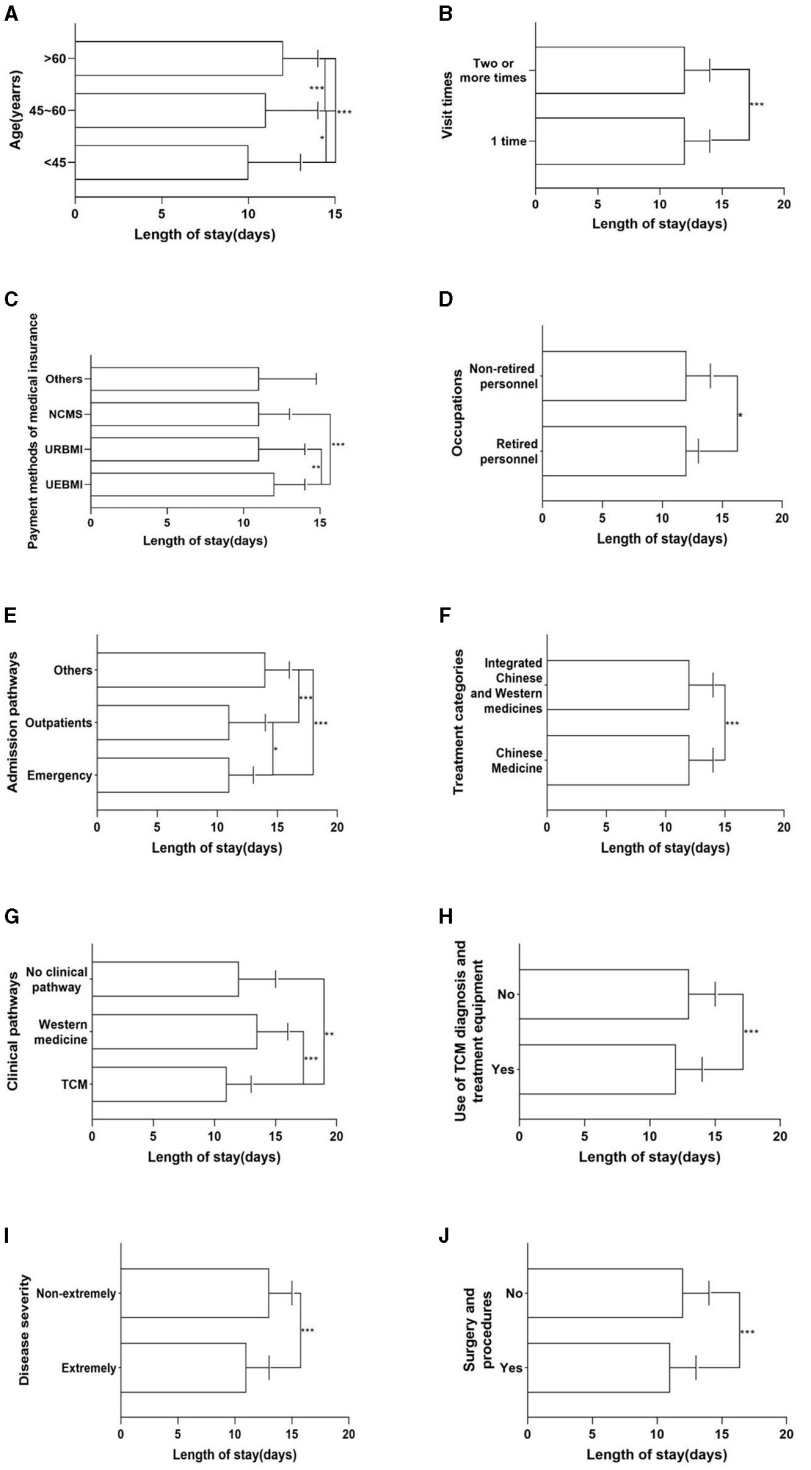
Univariate analysis results of length of stay. **(A)** Age. **(B)** Visit times. **(C)** Payment methods of medical insurance. **(D)** Occupations. **(E)** Admission pathways. **(F)** Treatment categories. **(G)** Clinical pathways. **(H)** Use of TCM diagnosis and treatment equipment. **(I)** Disease severity. **(J)** Surgery and procedures. *p < 0.05; **p < 0.01; ***p < 0.001.

**Figure 2:**
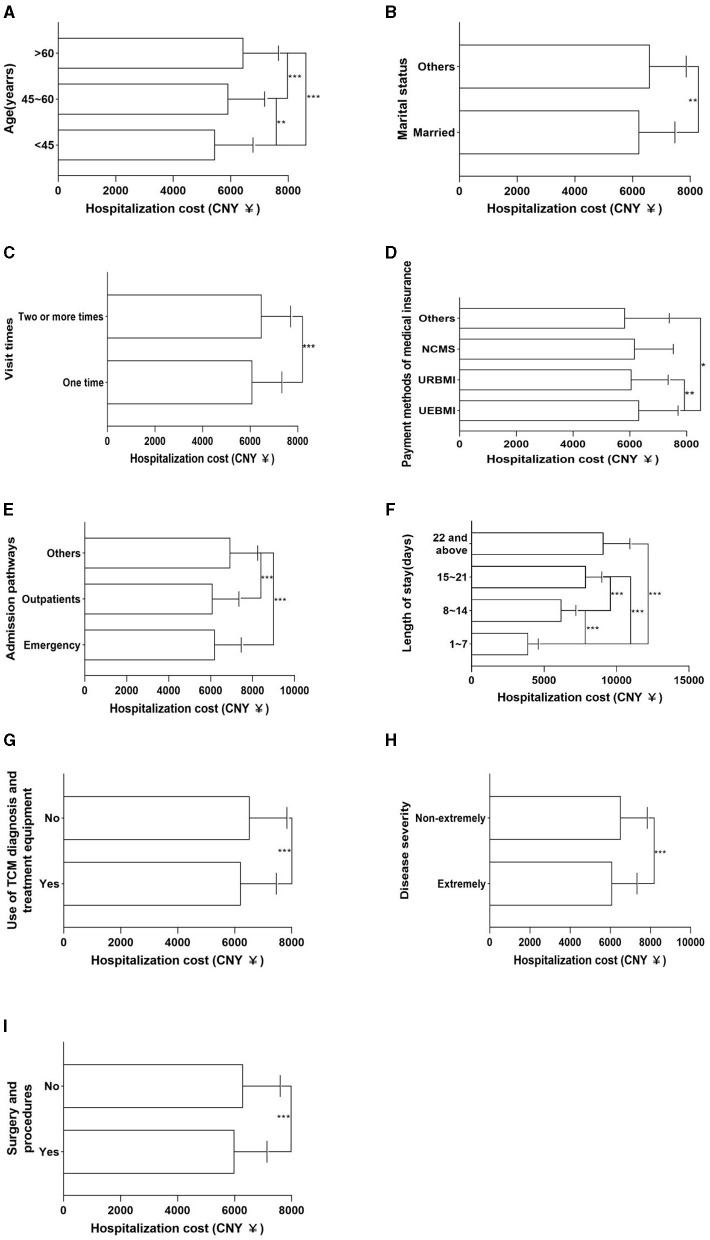
Univariate analysis results of hospitalization cost. **(A)** Age. **(B)** Marital status. **(C)** Visit times. **(D)** Payment methods of medical insurance. **(E)** Admission pathways. **(F)** Length of stay. **(G)** Use of TCM diagnosis and treatment equipment. **(H)** Disease severity. **(I)** Surgery and procedures. *p < 0.05; **p < 0.01; ***p < 0.001.

As can be seen from Table 2, there were significant differences (*p* < 0.05) in the length of stay among hypertensive patients with different ages, visit times, payment methods of medical insurance, occupations, admission pathways, treatment categories, clinical pathways, use of TCM diagnosis and treatment equipment, disease severity, and whether surgery and procedures were performed, and more comparison of differences can be seen in Figure 1. Furthermore, there were also statistical differences (*p* < 0.05) in the hospitalization cost of hypertensive patients by different ages, marital status, visit times, payment methods of medical insurance, admission pathways, length of stay, use of TCM diagnosis and treatment equipment, disease severity, and whether surgery and procedures were performed, and more comparison of differences can be seen in Figure 2.

**Table 2:** Description of the study sample (length of stay and hospitalization cost).

**Variable names**	**Variable categories**	***N* (%)**	**Length of stay (days)**		**Hospitalization cost (CNY** ¥**)**	
			***M*** **(P**_25_**, P**_75_**)**^e^	***Z/H*** **-value** ^f^ **/** ***p*** **-value**	***M*** **(P**_25_**, P**_75_**)**	***Z/H*** **-value/** ***p*** **-value**
Gender	Male	1,727 (48.04%)	12 (9, 14)	−0.278/0.781	6,208.289 (4,991.133, 7,463.729)	−1.284/0.199
	Female	1,868 (51.96%)	12 (9, 14)		6,266.977 (5,081.495, 7,517.017)	
Nationality	Han	3,501 (97.39%)	12 (9, 14)	−1.070/0.285	6,245.893 (5,052.704, 7,479.793)	−0.630/0.528
	Other ethnic groups	94 (2.61%)	11 (9, 13)		6,304.742 (4,970.031, 7,745.187)	
Age (years)	< 45	182 (5.06%)	10 (9, 13)	28.622/ < 0.001	5,448.945 (4,738.820, 6,776.972)	81.317/ < 0.001
	45–60	1,184 (32.93%)	11 (9, 14)		5,917.072 (4,769.390, 7,181.638)	
	>60	2,229 (62.00%)	12 (10, 14)		6,441.065 (5,303.748, 7,659.316)	
Marital status	Married	3,461 (96.27%)	12 (9, 14)	−0.437/0.662	6,227.116 (5,022.163, 7,470.215)	−2.761/0.006
	Others	134 (3.73%)	12 (10, 13.25)		6,592.672 (5,581.839, 7,861.733)	
Visit times	One time	2,124 (59.08%)	12 (9, 14)	−4.681/ < 0.001	6,086.396 (4,835.231, 7,334.465)	−6.552/ < 0.001
	Two or more times	1,471 (40.92%)	12 (10, 14)		6,484.139 (5,330.546, 7,704.961)	
Payment methods of medical insurance	UEBMI^a^	2,309 (64.23%)	12 (10, 14)	27.940/ < 0.001	6,330.094 (5,159.746, 7,515.878)	12.178/0.007
	URBMI^b^	606 (16.86%)	11 (9, 14)		6,060.021 (4,834.210, 7,353.051)	
	NCMS^c^	596 (16.58%)	11 (9, 13)		6,180.217 (4,948.426, 7,535.548)	
	Others	84 (2.34%)	11 (8, 14.75)		5,834.281 (3,973.610, 7,395.272)	
Occupations	Retired personnel	1,385 (38.53%)	12 (9, 13)	−2.231/0.026	6,320.342 (5,060.134, 7,481.272)	−0.723/0.469
	Non-retired personnel	2,210 (61.47%)	12 (9, 14)		6,198.538 (5,035.872, 7,486.123)	
Admission pathways	Emergency	830 (23.09%)	11 (9, 13)	269.393/ < 0.001	6,201.436 (4,907.115, 7,457.158)	104.115/ < 0.001
	Outpatients	2,217 (61.67%)	11 (9, 14)		6,089.061 (4,896.043, 7,346.399)	
	Others	548 (15.24%)	14 (12, 16)		6,933.002 (5,793.101, 8,240.037)	
Treatment categories	Chinese medicine	2,374 (66.04%)	12 (9, 14)	−3.395/0.001	6,260.372 (5,077.969, 7,469.626)	−0.375/0.708
	Integrated Chinese and Western medicines	1,221 (33.96%)	12 (9, 14)		6,218.038 (4,956.771, 7,533.129)	
Length of stay (days)	1–7	453 (12.60%)	6 (5, 7)	2,404.479/ < 0.001	3,901.713 (3,249.464, 4,605.480)	1,281.254/ < 0.001
	8–14	2,483 (69.07%)	11 (10, 13)		6,196.272 (5,256.654, 7,198.669)	
	15–21	611 (17.00%)	16 (15, 17)		7,880.837 (6,969.960, 8,984.650)	
	22 and above	48 (1.34%)	23 (22, 25)		9,101.600 (8,317.079, 1,0924.638)	
Clinical pathways	Chinese medicine	2,471 (68.73%)	11 (9, 13)	62.088/ < 0.001	6,207.420 (5,069.242, 7,400.979)	5.722/0.057
	Western medicine	28 (0.78%)	13.5 (12, 16)		6,832.700 (5,439.879, 8,619.730)	
	No clinical pathway	1,096 (30.49%)	12 (10, 15)		6,331.794 (4,942.251, 7,742.514)	
Use of TCM^*d*^ preparations	Yes	1,880 (52.29%)	12 (9, 14)	−0.958/0.338	6,245.983 (5,138.758, 7,524.630)	−1.008/0.313
	No	1,715 (47.71%)	12 (9, 14)		6,246.410 (4,970.603, 7,455.126)	
Use of TCM^*d*^ diagnosis and treatment equipment	Yes	3,263 (90.76%)	12 (9, 14)	−4.582/ < 0.001	6,211.532 (5,018.061, 7,459.125)	−2.992/0.003
	No	332 (9.24%)	13 (10, 15)		6,526.797 (5,378.767, 7,832.870)	
Use of TCM^*d*^ diagnosis and treatment techniques	Yes	3,512 (97.69%)	12 (9, 14)	−0.447/0.655	6,252.858 (5,052.493, 7,480.795)	−0.640/0.522
No	83 (2.31%)	12 (9, 14)		5,866.920 (4,900.576, 7,730.989)	
Disease severity	Extremely high risk	2,473 (68.79%)	11 (9, 13)	−14.290/ < 0.001	6,090.096 (4,894.673, 7,333.047)	−7.695/ < 0.001
	Non-extremely high risk	1,122 (31.21%)	13 (10, 15)		6,523.289 (4,894.673, 7,842.733)	
Surgery and procedures	Yes	773 (21.50%)	11 (9, 13)	−7.148/ < 0.001	5,992.718 (4,805.164, 7,134.159)	−5.431/ < 0.001
	No	2,822 (78.50%)	12 (9, 14)		6,291.816 (5,146.004, 7,595.878)	

^a^UEBMI, the urban employee basic medical insurance system; ^b^URBMI, the urban residents' basic medical insurance system; ^c^NCMS, the new cooperative medical scheme; ^d^TCM, traditional Chinese medicine; ^e^M (P_25_, P_75_), median (the first quartile, the third quartile); ^f^Z/H-value, Mann–Whitney *U*-test statistical value or Kruskal–Wallis *H*-test statistical value.

### 3.2 Multivariate linear regression results of length of stay and hospitalization cost

Results of multivariate linear regression showed that the length of stay of hypertensive patients was affected by age, visit times, payment methods of medical insurance, other ways of admission pathways, and disease severity (*p* < 0.05), and the regression equation of length of stay (*F* = 20.322, *p* < 0.001, *R*^2^ = 0.078) was as follows:

***Y*_1_** **=** **0.953** **+** 0.037^*^X_3 − 1_ **+** 0.042^*^X_3 − 2_ **+** 0.032^*^X_5_ – 0.020^*^X_6 − 1_ – 0.027^*^X_6 − 2_ **+** 0.015^*^X_8 − 1_ **+** 0.098^*^X_8 − 2_ – 0.028^*^X_14_.

Hospitalization cost was affected by age, marital status, payment methods of medical insurance, admission pathways, surgery and procedures, and length of stay (*p* < 0.05), and the regression equation of hospitalization cost (*F* = 327.863, *p* < 0.001, *R*^2^ = 0.543) was as follows:

***Y*_2_** **=** **3.103** **+** 0.038^*^X_3 − 2_ **+** 0.019^*^M*X*_4_ **+** 0.015^*^X_6 − 2_ – 0.012^*^X_8 − 1_ – 0.010^*^X_15_ **+** 0.610^*^Y_1_.

The results are summarized in Table 3. Further calculation based on analysis results showed that the residual path coefficient was 0.676 (*P*_*e*_ = $\sqrt{1-R^{2}}$ = 0.676), which is lower than the standardized coefficient of *Y*_1_, indicating that there are other influencing factors existing as well. Path models can be developed based on the results of multiple linear regressions to explore other influences on hospitalization cost comprehensively.

**Table 3:** Multiple linear regression results of the length of stay and hospitalization cost of hypertension patients.

**Variables**	**Log (length of stay)**				**Log (hospitalization cost)**			
	***B** ^e^*	**Beta** ^f^	***t-*** **value**	***p-*** **value**	***B***	**Beta**	***t-*** **value**	***p-*** **value**
Constant	0.953	—	35.476	< 0.001	3.103	—	180.744	< 0.001
**Age (ref** = < **45)**								
45–60	0.037	0.100	2.741	0.006	0.013	0.040	1.583	0.113
>60	0.042	0.116	3.023	0.003	0.038	0.123	4.773	< 0.001
Marital status	—	—	—	—	0.019	0.024	2.069	0.039
Visit times	0.032	0.089	5.133	< 0.001	0.006	0.021	1.707	0.088
**Payment methods of medical insurance (ref** = **URRBMI**^a^**)**								
URBMI^b^	−0.020	−0.042	−2.254	0.024	−0.003	−0.009	0.733	0.464
NCMS^c^	−0.027	−0.057	−2.910	0.004	0.015	0.037	3.062	0.002
Others	−0.034	0.030	−1.813	0.070	−0.021	−0.021	−1.850	0.064
Occupation	0.003	0.008	0.349	0.727	—	—	—	—
**Admission pathways (ref** = **emergency)**								
Outpatients	0.015	0.042	2.084	0.037	−0.012	−0.039	−2.802	0.005
Others	0.098	0.200	7.238	< 0.001	−0.010	−0.025	−1.419	0.156
Treatment category	0.012	0.033	1.784	0.075	—	—	—	—
**Clinical pathway (ref** = **Chinese medicine)**								
Western medicine	0.046	0.023	1.414	0.157	—	—	—	—
No clinical pathway	−0.012	−0.031	−1.672	0.095	—	—	—	—
Use of TCM^d^ diagnosis and treatment equipment	−0.010	−0.017	−1.017	0.309	−0.002	−0.004	−0.321	0.748
Disease severity	−0.028	−0.073	−2.827	0.005	−0.001	−0.004	−0.272	0.785
Surgery and procedures	−0.014	−0.032	−1.780	0.075	−0.010	−0.029	−22.323	0.020
Log (length of stay)	—	—	—	—	0.610	0.721	61.342	< 0.001
*R^2^*-value	0.078				0.543			
*F*-value	20.322				327.863			
*P*-value	< 0.001				< 0.001			

^a^UEBMI, the urban employee basic medical insurance system; ^b^URBMI, the urban residents' basic medical insurance system; ^c^NCMS, the new cooperative medical scheme; ^d^TCM, traditional Chinese medicine; ^e^*B*, unstandardized coefficients; ^f^Beta, standardized coefficients.

### 3.3 Path analysis results of hospitalization cost

**Figure 3:**
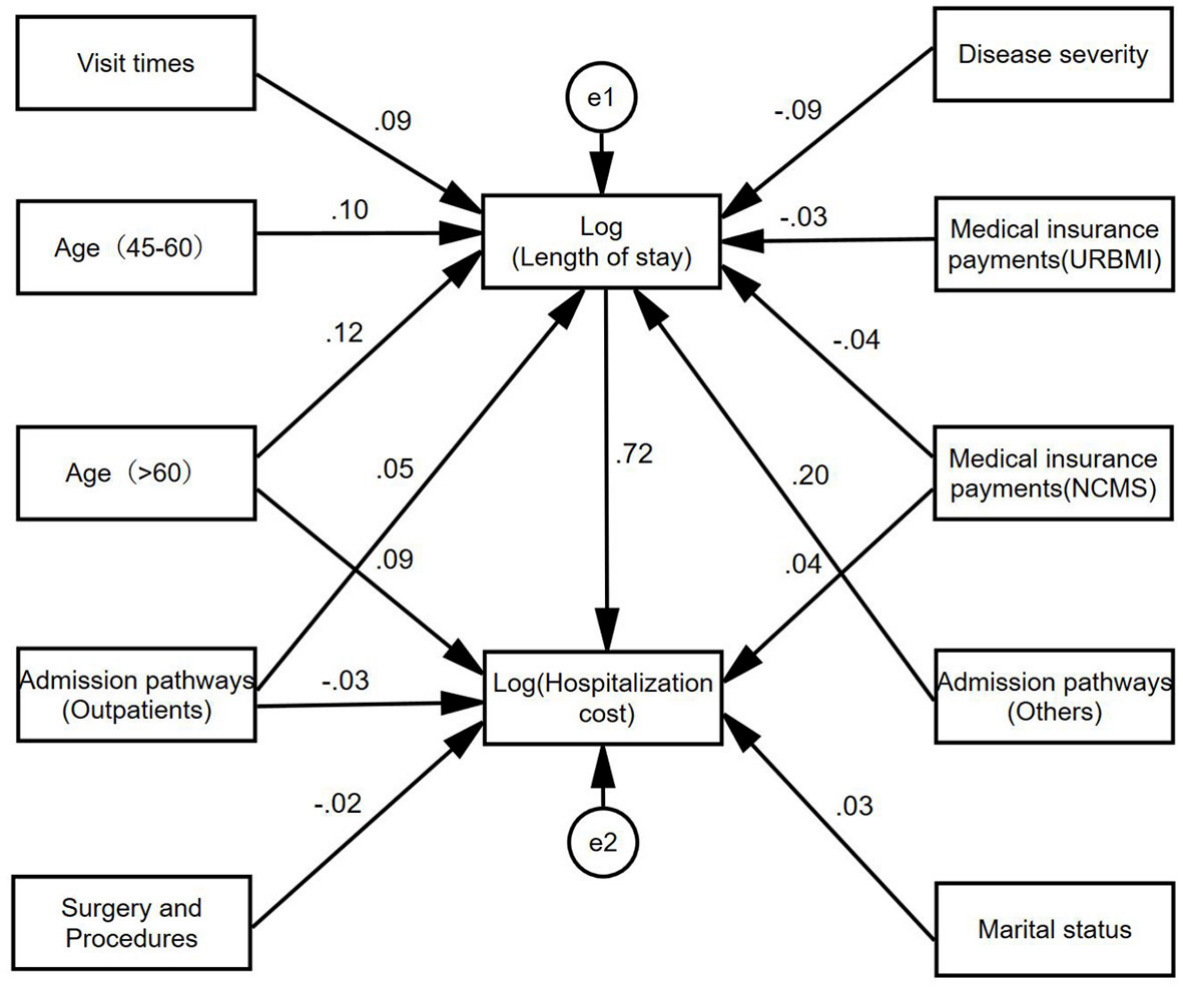
Path diagram of influencing factors of hospitalization cost of hypertension patients.

As shown in Figure 3, visit times, age (45–60), admission pathways (Outpatient, Others), disease severity, and payment methods of medical insurance could indirectly affect the hospitalization cost through the length of stay. In addition, age (>60), admission pathways (Outpatients), marital status, payment methods of medical insurance (NCMS), and surgery and procedures could also affect the hospitalization cost directly.

Based on the path model, the effects decomposition of the influencing factors of hospitalization cost could be derived, and the specific results were summarized in Table 4. The effect of each factor on hospitalization cost ranked as follows: length of stay, age (>60), admission pathways (Others), payment methods of medical insurance (NCMS), visit times, disease severity, admission pathways (Outpatients), marital status, payment methods of medical insurance (URBMI), surgery and procedures.

**Table 4:** Effect decomposition table of influencing factors of hospitalization cost of hypertension patients (tertiary).

**Variables**	**Direct effect**	**Indirect effect**	**Total effect**	**Ranking of total effect**
Age (45–60)	–	0.074	0.074	4
Age (> 60)	0.092	0.066	0.158	2
Marital status	0.028	–	0.028	9
Visit times	–	0.064	0.064	6
Payment methods of medical insurance (URBMI^a^)	–	−0.024	−0.024	10
Payment methods of medical insurance (NCMS^b^)	0.041	0.030	0.071	5
Admission pathways (Outpatients)	−0.029	−0.021	−0.05	8
Admission pathways (Others)	–	0.144	0.144	3
Disease severity	–	−0.063	−0.063	7
Surgery and procedures	−0.024	–	−0.024	11
Length of stay	0.722	–	0.722	1

^a^URBMI, the urban residents basic medical insurance system; ^b^NCMS, the new cooperative medical scheme.

## 4 Discussion

Current research on the cost of hypertension mainly focuses on comprehensive (Western medicine) hospitals, while our study is based on the TCM hospitals' official database from local governments in China, with rigorous and appropriate statistical methods to analyze the influence factors on hospitalization cost of hypertensive patients. As a result, this study will powerfully enrich and expand the content of the current research on cost control of hypertension, promote the development of health economics with Chinese characteristics, and be of great practical significance in giving full play to the advantages of traditional Chinese medicine in the treatment of chronic diseases such as hypertension and the reduction of the economic burden of disease in China.

Through the research, we found the length of stay of male patients with hypertension in TCM hospitals is basically equal to female patients, while the hospitalization cost of male patients is slightly higher than female patients. What's more, the age of patients is a stronger factor on hospitalization cost, which increases with growing age. What made this possible is that the older adult due to the continuous aging of the patient's organism and decreasing resistance, have a higher risk of disease and are prone to repeat and prolonged hospitalization, which consumes more healthcare resources (35, 36). Additionally, patients admitted by other pathways, such as medical referral and cross-province medical treatment, will significantly increase hospitalization cost (37). The fact that medical insurance policies of some insured places have set up differential treatment for medical referral and cross-provincial medical treatment so that their reimbursement ratio is lower than other groups, is the main reason for the current Chinese government's continuous implementation of medical insurance reform. In terms of visit times, patients with repeat visit may have relatively complex and severe conditions that show a tendency toward poorer outcomes, of which hospitalization cost will also be relatively high. As the condition progressed to critical, the risk of increased hospitalization cost rose dramatically, implying a greater possibility of resuscitation, and further increasing the risk of higher hospitalization cost.

In complete harmonization with existing studies, hospitalization cost is higher for older patients, the greater the number of visit times, and longer length of stay (38–40). Meanwhile, we found the length of stay and hospitalization cost are higher among non-retired personnel patients (41–43), which may be due to the fact that higher work pressure induces more serious hypertensive disorders with more hospitalization cost (44–46). This result coincided with multiple existing studies (47, 48), which indicate that young and middle-aged working hypertension patients tend to have a predominantly cerebral workload, older retired hypertension patients are mostly engaged in physical work, as well as working mental work face more pressure, whose prevalence of hypertension and severity of the disease also increase continuously. In addition, patients with hypertension admitted through other pathways have longer length of stay and higher hospitalization cost, mainly because patients' conditions are more complex or severe with more special investigations and treatments. At the same time, the referral pathway may result in poor transfer of information between multiple providers, which increases duplication of tests and treatments and further increases hospitalization cost (49, 50).

Confusingly, hypertensive patients with more critical diseases and undergoing surgeries and procedures had fewer length of stay and lower hospitalization cost than patients with non-critical diseases and without surgeries and procedures. Combined with the characteristics of TCM syndrome differentiation and treatment, the explanations conjecture are as follows: Firstly, the more critical the disease, the more standardized the treatment, surgical operation, and medical service process are performed. Subsidiary, surgery and procedures in the sense of TCM, such as acupuncture, moxibustion, cupping therapy, tuina, qigong, and other external treatment methods, have always been safer, more convenient, and less costly than Western surgeries (51–53), which reduces the length of stay and hospitalization cost with excellent therapeutic results. Moreover, according to the relevant policies of Chinese medical insurance, and surgery and procedures will receive greater reimbursement support from medical insurance, so the actual hospitalization cost is relatively low.

In general, in addition to the length of stay and age, the hospitalization cost of hypertension patients in TCM hospitals is also affected by the admission pathways, payment methods of medical insurance, and visit times, among which the length of stay has the greatest impact on the hospitalization cost of patients with hypertension (5, 54, 55). The longer the length of stay, the greater the medical-economic pressure the patients face. Notably, the health literacy level of hypertensive patients also affects hospitalization cost (56). Health literacy affects patients' lifestyle, medication adherence treatment behavior, etc., which in turn affects the disease management and prognosis of patients with chronic diseases (57–59). Some studies have shown that the proportion of hypertension patients with health literacy was 10.10%, which was lower than 16.13% of the general population (60), indicating that improving health literacy is an important strategy to prevent and control hypertension and other chronic diseases.

## 5 Conclusions

In this study, 3,595 inpatients with a primary diagnosis of tertiary hypertension in Tianshui Hospital of Traditional Chinese Medicine, Gansu Province, China from January 2017 to June 2022 were selected as the research objects, to explore the influencing factors of hospitalization cost of hypertension patients. From our analysis results, the hospitalization cost of hypertensive patients is mainly affected by length of stay, age, admission pathways, payment methods of medical insurance, and visit times, and the length of stay is the most important and core influencing factor. As chronic diseases such as hypertension intensify and threaten the quality of life and health of all human beings, the Chinese government should continue to optimize the methods of diagnosis and treatment of TCM, incorporate the “combining disease and syndrome” into the process of treating illnesses to classify and define treatments, and actively exert the characteristics and advantages of TCM in the treatment of chronic diseases such as hypertension. Meanwhile, the China government should continue to deepen the reform of medical insurance policies, enhance the strength of outpatient medical insurance for chronic diseases, and optimize the structure of medical insurance treatment for patients with chronic diseases. More importantly, the China government should innovate a compensation mechanism for TCM to incentivize patients to actively use TCM to improve their health literacy, thereby reducing the length of stay, relieving their pain, and lowering the burden of medical costs.

## Data availability statement

The raw data supporting the conclusions of this article will be made available by the authors, without undue reservation.

## Author contributions

H-jH: Conceptualization, Data curation, Formal analysis, Methodology, Project administration, Software, Writing – original draft, Writing – review & editing. T-zC: Investigation, Validation, Writing – review & editing. YC: Formal analysis, Validation, Writing – review & editing. Y-hB: Validation, Writing – original draft. M-eC: Software, Writing – original draft. J-yY: Resources, Supervision, Writing – review & editing. Z-hL: Conceptualization, Funding acquisition, Resources, Supervision, Writing – review & editing.
